# Calcitonin controls bone formation by inhibiting the release of sphingosine 1-phosphate from osteoclasts

**DOI:** 10.1038/ncomms6215

**Published:** 2014-10-21

**Authors:** Johannes Keller, Philip Catala-Lehnen, Antje K. Huebner, Anke Jeschke, Timo Heckt, Anja Lueth, Matthias Krause, Till Koehne, Joachim Albers, Jochen Schulze, Sarah Schilling, Michael Haberland, Hannah Denninger, Mona Neven, Irm Hermans-Borgmeyer, Thomas Streichert, Stefan Breer, Florian Barvencik, Bodo Levkau, Birgit Rathkolb, Eckhard Wolf, Julia Calzada-Wack, Frauke Neff, Valerie Gailus-Durner, Helmut Fuchs, Martin Hrabĕ de Angelis, Susanne Klutmann, Elena Tsourdi, Lorenz C. Hofbauer, Burkhard Kleuser, Jerold Chun, Thorsten Schinke, Michael Amling

**Affiliations:** 1 Department of Osteology and Biomechanics, University Medical Center Hamburg-Eppendorf, 20246 Hamburg, Germany; 2 Institute of Human Genetics, University Hospital Jena, 07743 Jena, Germany; 3 Institute of Nutritional Science, University of Potsdam, 14469 Potsdam, Germany; 4 Center for Molecular Neurobiology, University of Hamburg, 20251 Hamburg, Germany; 5 Institute of Clinical Chemistry, University Medical Center Hamburg-Eppendorf, 20246 Hamburg, Germany; 6 Institute of Pathophysiology, University Hospital Essen, 45122 Essen, Germany; 7 Institute of Molecular Animal Breeding and Biotechnology, Gene Center, Ludwig-Maximilian-University, 81377 Munich, Germany; 8 German Mouse Clinic, Institute of Experimental Genetics, Helmholtz Center Munich, 85764 Neuherberg, Germany; 9 German Mouse Clinic, Institute of Pathology, Helmholtz Center Munich, 85764 Neuherberg, Germany; 10 Center of Life and Food Sciences Weihenstephan, Technische Universität München, 85354 Freising, Germany; 11 German Center for Diabetes Research, 85764 Neuherberg, Germany; 12 Department of Nuclear Medicine, University Medical Center Hamburg-Eppendorf, 20246 Hamburg, Germany; 13 Department of Medicine III, Dresden University Medical Center, 01307 Dresden, Germany; 14 Scripps Research Institute, La Jolla, California 92937, USA

## Abstract

The hormone calcitonin (CT) is primarily known for its pharmacologic action as an inhibitor of bone resorption, yet CT-deficient mice display increased bone formation. These findings raised the question about the underlying cellular and molecular mechanism of CT action. Here we show that either ubiquitous or osteoclast-specific inactivation of the murine CT receptor (CTR) causes increased bone formation. CT negatively regulates the osteoclast expression of *Spns2* gene, which encodes a transporter for the signalling lipid sphingosine 1-phosphate (S1P). CTR-deficient mice show increased S1P levels, and their skeletal phenotype is normalized by deletion of the S1P receptor S1P_3_. Finally, pharmacologic treatment with the nonselective S1P receptor agonist FTY720 causes increased bone formation in wild-type, but not in S1P_3_-deficient mice. This study redefines the role of CT in skeletal biology, confirms that S1P acts as an osteoanabolic molecule *in vivo* and provides evidence for a pharmacologically exploitable crosstalk between osteoclasts and osteoblasts.

## Abstract (web summary)

The regulatory role of calcitonin in bone homeostasis is well studied, yet its molecular activity is poorly understood. The authors show that calcitonin regulates bone cells function by inhibiting the osteoclast secretion of sphingosine 1-phosphate, a lipid mediator of osteoclast–osteoblast crosstalk.

## Graphical abstract

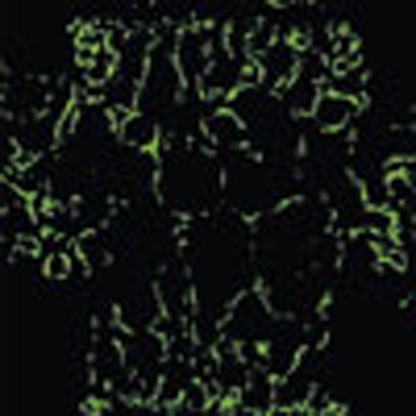

Osteoporosis represents a major public health problem, not only because of its high prevalence, but also because osteoporotic fractures are associated with high morbidity and mortality. At the cellular level, osteoporosis is explained by imbalanced bone remodelling, a physiologically relevant homeostatic process mediated through bone-resorbing osteoclasts and bone-forming osteoblasts^1^. On the basis of their cellular differences, there are two distinct therapeutic options to treat osteoporosis, either osteoclast inhibition (anti-resorptive) or osteoblast activation (osteoanabolic). Currently, the vast majority of patients are medicated by anti-resorptive means, and there is only one osteoanabolic treatment option so far, daily injection of parathyroid hormone (PTH) or a PTH fragment^2^. Since a long-term blockade of bone remodelling may have adverse effects on skeletal integrity, one of the major goals of skeletal research is to identify novel target proteins for osteoanabolic medication.

One of the hormones considered to influence bone remodelling is calcitonin (CT), which was discovered more than 50 years ago as a calcium-lowering factor produced by thyroidal C cells^3^,^4^. CT is proteolytically released from procalcitonin (PCT), representing a human sepsis biomarker potentially enhancing sepsis severity^5^,^6^. CT has been shown to exert its effects through the CT receptor (CTR), a serpentine protein expressed at high levels in kidney and hypothalamus^7^,^8^. In bone, CT exclusively binds to osteoclasts, exhibiting the highest CTR-density, and causes cessation of their activity. This anti-resorptive effect is especially pronounced in studies employing salmon CT, displaying a 50-fold higher potency than mammalian CT^9^,^10^. On the basis of these findings, CT was considered to represent the functional counterpart to PTH, which physiologically activates bone resorption by altering gene expression in osteoblasts.

Although the pharmacologic actions of CT have been studied extensively, there is a marked uncertainty about its role in mammalian physiology^11^,^12^. This is primarily based on the fact that patients with CT deficiency following thyroidectomy do not display the expected osteoporosis, and that bone mineral density was found decreased in individuals with medullary thyroid carcinoma (MTC)^10^,^13^. In addition, mice lacking *Calca*, the gene encoding CT and its related family member alpha CT-gene-related peptide (αCGRP), unexpectedly displayed high bone mass, in contrast to mice specifically lacking αCGRP^14^,^15^,^16^. Importantly, although bone resorption was increased in aged *Calca*-deficient mice, there was a twofold higher bone formation rate at all ages analysed suggesting that the primary physiological function of CT is to limit osteoblast activity. These findings did not only challenge our understanding of CT as a bone-preserving hormone, they also raised several questions of key significance. In addition, they suggested that understanding the cellular and molecular mode of CT action would help to identify novel osteoanabolic treatment options.

To study the impact of CT on skeletal integrity, we generated a mouse model allowing cell type-specific deletion of two *Calcr* exons encoding the CT-binding site of the CTR. Here we show that CTR inactivation in all cell types or in osteoclasts specifically causes increased bone formation. The indirect influence of CT on bone formation is molecularly explained by a CTR-mediated inhibition of *Spns2* expression, encoding a transporter for sphingosine 1-phosphate (S1P), one of the previously identified molecules potentially coupling bone formation to bone resorption^17^,^18^. Likewise, S1P levels are increased in bones of CTR-deficient mice, and their skeletal phenotype is normalized by additional absence of S1P_3_, a S1P receptor expressed by osteoblasts.

## Results

### Inactivation of the CTR specifically affects bone formation

**Figure 1:**
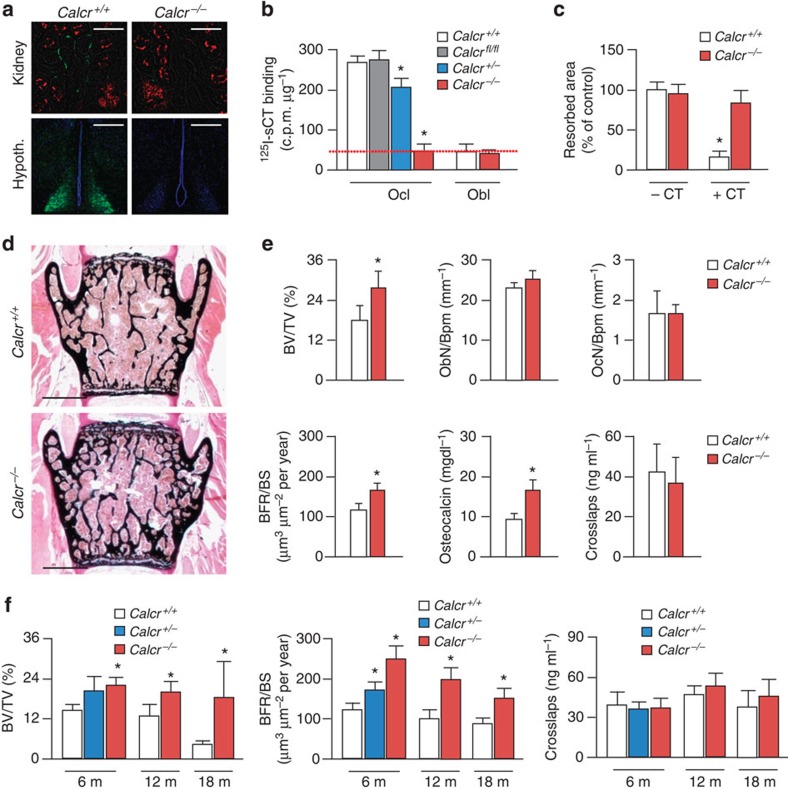
Inactivation of the CTR specifically increases osteoblast function. (**a**) Immunohistochemistry for the CTR (stained in green) in kidney and hypothalamus (hypoth.) sections from wild-type and *Calcr*^−/−^ mice. Scale bars, 50 μm (top) and 200 μm (bottom). (**b**) Binding of ^125^I-salmon CT to osteoclasts or osteoblasts of the indicated genotypes. The dotted red line represents nonspecific binding; *n*=3 cultures per group. *P<0.05 versus wild-type. Data are representative of two independent experiments. (**c**) Quantification of dentin resorption by wild-type and *Calcr*^−*/*−^ osteoclasts differentiated in the absence or presence of CT; *n*=3 cultures per group. *P<0.05 versus control. Data are representative of three independent experiments. (**d**) Von Kossa/van Gieson-staining of non-decalcified spine sections from 3-month-old female wild-type and *Calcr*^−*/*−^ mice (scale bars, 1 mm). (**e**) Histomorphometric quantification of the trabecular bone volume per tissue volume (BV/TV), the osteoblast number per bone perimeter (ObN/Bpm), the osteoclast number per bone perimeter (OcN/Bpm) and the bone formation rate per bone surface (BFR/BS). Concentrations of osteocalcin and collagen degradation products (crosslaps) were measured in serum; *n*=6 mice per group. *P<0.05 versus wild-type. (**f**) Histomorphometric assessment of BV/TV, BFR/BS and serum crosslaps in 6-, 12- and 18-month-old female wild-type and *Calcr*^*−/−*^ mice, and of 6-month-old *Calcr*^*+/−*^ mice; *n*=6 mice per group. *P<0.05 versus wild-type. All error bars indicate s.d. *P* values were assessed by two-tailed Student’s *t*-test.

To understand the mechanism of CT action on bone remodelling, we generated a floxed allele of the CTR by homologous recombination in embryonic stem cells. As it was reported that deletion of exons 6 and 7 from the *Calcr* gene causes embryonic lethality^19^, we constructed a targeting vector resulting in the insertion of loxP sites 5′ of exon 6 and 3′ of exon 7, respectively (Supplementary Fig. 1a). After confirming homologous recombination by Southern blotting (Supplementary Fig. 1b), we removed the neomycin resistance cassette by Flp-mediated recombination and injected embryonic stem cells carrying one floxed *Calcr* allele into blastocysts to generate heterozygous mice. These were first mated with *CMV-Cre* transgenic mice^20^, leading to the generation of mice carrying a recombined *Calcr* allele, independent of *Cre* expression. The subsequent mating of *Calcr*^*+/−*^ mice resulted in wild-type, heterozygous and *Calcr*-deficient animals, which were born at the expected Mendelian ratio (Supplementary Fig. 1c,d). Since this result did not confirm the embryonic lethality of *Calcr*-deficient mice observed by others^19^, it was important to demonstrate loss of a functional CTR in our model. Immunohistochemistry confirmed the absence of the CTR protein in kidney and hypothalamus sections from *Calcr*^−/−^ mice (Fig. 1a), and binding assays employing iodinated CT demonstrated the lack of specific binding to cultured *Calcr*^−/−^ osteoclasts (Fig. 1b). In addition, CT inhibited the resorptive activity of wild-type, but not of *Calcr*^*−/−*^ osteoclasts, when these cells were cultured on dentin chips for 10 days (Fig. 1c).

After backcrossing the mutant allele into the C57Bl/6 genetic background we analysed 12 weeks old wild-type and *Calcr*^*−/−*^ littermates for potential phenotypic abnormalities. Determination of blood parameters demonstrated no alterations in mineral homeostasis (Supplementary Table 1) and ruled out the existence of hepatic (Supplementary Table 2) and renal defects (Supplementary Table 3) in *Calcr*^−/−^ mice, which was confirmed by histology (Supplementary Fig. 1e). Importantly, *Calcr*^−/−^ mice displayed higher circulating levels of alkaline phosphatase, suggesting increased bone formation as an underlying cause. Therefore, we went on to analyse the skeletal phenotype of *Calcr*^−/−^ mice using non-decalcified histology followed by static and dynamic histomorphometry. Here we observed an increased trabecular bone mass compared with wild-type littermates, which was solely explained by increased bone formation (Fig. 1d,e). Since osteoporosis is most common in aged individuals, we next assessed the skeletal phenotype of *Calcr*^−/−^ mice at 6, 12 and 18 months of age. Compared with wild-type littermates *Calcr*^−/−^ mice displayed an increased trabecular bone mass together with an elevated bone formation rate at all ages analysed (Fig. 1f). In contrast, bone resorption was not increased in *Calcr*^−/−^ mice, representing an unexpected finding as our previous analysis of aged CT-deficient mice revealed a high bone turnover phenotype with a more than twofold increase in bone resorption markers^15^.

**Figure 2:**
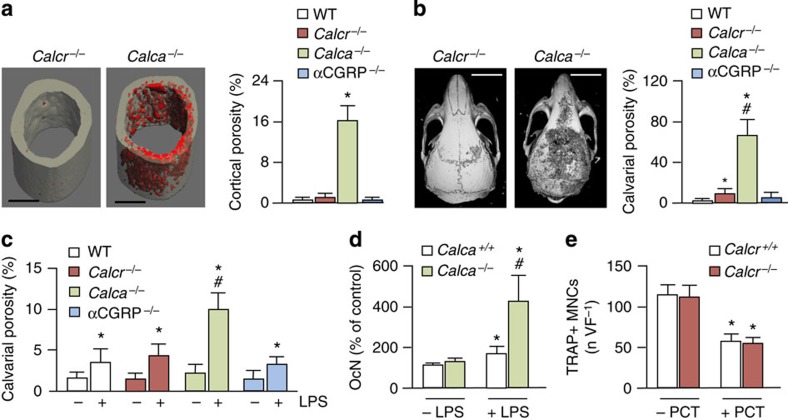
Phenotypic differences between *Calcr*^*−/−*^ and *Calca*^*−/−*^ mice. (**a**) μCT scans of the femur from 18-month-old female *Calcr*^*−/−*^ and *Calca*^*−/−*^ mice. Scale bar, 500 μm. Cortical porosity for mice of the indicated genotypes is shown on the right; *n*=5. *P<0.05 versus wild-type. (**b**) μCT scans of the calvaria from 18-month-old female *Calcr*^*−/−*^ and *Calca*^*−/−*^ mice. Scale bar, 1 mm. Calvarial porosity for mice of the indicated genotypes is shown on the right; *n*=5. *P<0.05 versus wild-type. (**c**) Calvarial porosity induced by local LPS injection in 6 weeks old wild-type, *Calcr*^*−/−*^, *Calca*^*−/−*^ and αCGRP^*−/−*^ mice; *n*=5. *P<0.05 versus control. #*P*<0.05 versus wild-type. (**d**) Osteoclast numbers in wild-type and *Calca*^*−/−*^ mice with or without LPS treatment; *n*=5. *P<0.05 versus control. #*P*<0.05 versus wild-type. (**e**) TRAP-positive multinucleated cells (TRAP^+^MNCs) per visual field (VF) after differentiation of bone marrow cells with M-CSF and RANKL in the presence or absence of 10^*−*8^ M PCT; *n*=3; *P<0.05 versus non-treated wild-type. Data are representative of two independent experiments. All error bars indicate s.d. *P* values were assessed by two-tailed Student’s *t*-test.

To confirm this striking difference between the two models we applied μCT-scanning and determined porosity of the femoral cortex (Fig. 2a) and calvarial bone (Fig. 2b) in 18-month-old mice. While only a moderate difference between wild-type and *Calcr*^−/−^ mice could be detected, we found severe cortical and calvarial porosity in CT-deficient *Calca*^−/−^ mice that were maintained on the same genetic background. Since αCGRP^−/−^ mice did not display this phenotype either, we hypothesized that the observed differences between *Calcr*^−/−^ and *Calca*^−/−^ mice are explained by the fact that the latter ones additionally lack procalcitonin (PCT), a well-established sepsis biomarker, which has been shown to interact with receptor complexes other than CT^6^,^21^. To address this possibility we injected lipopolysaccharide (LPS) onto the calvaria of 6-week-old wild-type, *Calcr*^−/−^, *Calca*^−/−^ and αCGRP^−/−^ mice and applied μCT scanning 1 week thereafter (Fig. 2c). As expected, LPS treatment led to a significant increase in calvarial porosity in mice of all genotypes, yet its effect was significantly more pronounced in *Calca*^−/−^ mice. At the histological level these differences were explained by increased osteoclastogenesis (Fig. 2d), which led us to analyse the effect of PCT on osteoclast formation *ex vivo*. Here we found that the number of TRAP-positive multinucleated osteoclasts was significantly reduced by PCT, and most importantly the same was the case in *Calcr*^−/−^ cultures (Fig. 2e). Collectively, these findings do not only suggest that PCT can affect bone mass in a CTR-independent manner, they also demonstrated that the major physiological function of the CTR is to limit the activity of osteoblasts.

### The CTR in osteoclasts controls bone formation

**Figure 3:**
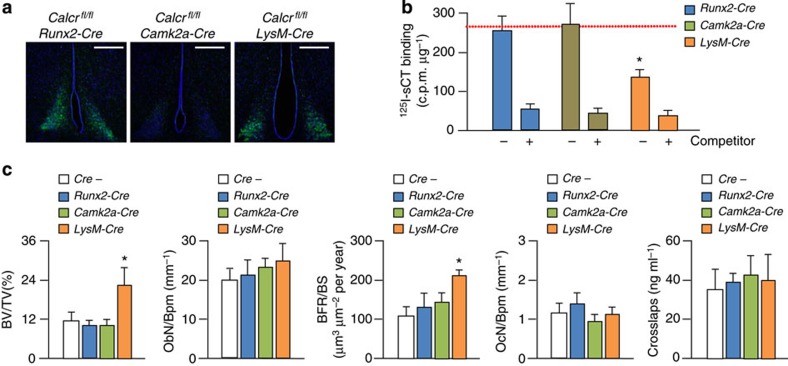
Control of bone formation by the CTR in osteoclasts. (**a**) Immunohistochemistry for the presence of the CTR in hypothalamus sections. Scale bar, 200 μm. (**b**) Binding of ^125^I-sCT to differentiated osteoclasts derived from mice of the indicated genotypes. The dotted red line represents binding to *Calcr*^*fl/fl*^ osteoclasts; *n*=3 cultures per group. *P<0.05 versus *Calcr*^*fl/fl*^ osteoclasts. Data are representative of two independent experiments. (**c**) Quantification of BV/TV, ObN/Bpm, BFR/BS, OcN/Bpm and measurement of serum crosslaps in 12-month-old female mice of the genotypes indicated above; *n*≥5 mice per group. *P<0.05 versus *Calcr*^*fl/fl*^ mice. All error bars indicate s.d. *P* values were assessed by two-tailed Student’s *t*-test.

To identify the relevant expression site mediating the inhibitory influence of the CTR on bone formation, we crossed *Calcr*^*fl/fl*^ mice with transgenic mice expressing the Cre recombinase either in osteoblasts (*Runx2-Cre*), in the hypothalamus (*CamK2a-Cre*) or in the osteoclast lineage (*Lysm-Cre*)^22^,^23^,^24^. The specificity of the chosen approach was confirmed by genomic PCR amplifying the recombined allele from different tissues and bone cells (Supplementary Fig. 2a). In addition, we performed immunohistochemistry on hypothalamus sections (Fig. 3a) and binding assays with cultured osteoclasts (Fig. 3b), demonstrating that *Calcr*^*fl/fl*^ mice carrying the *CamK2a-Cre* transgene lacked the CTR only in the hypothalamus, whereas binding of iodinated CT was decreased only in osteoclasts derived from *Calcr*^*fl/fl*^ mice carrying the *Lysm-Cre* transgene. We next analysed the skeletal phenotype of the corresponding mice at 6 months (Supplementary Fig. 2b) and 12 months (Fig. 3b) of age and found that the trabecular bone volume was increased only in *Calcr*^*fl/fl*^ mice carrying the *Lysm-Cre* transgene. Consistent with the results from *Calcr*^−/−^ mice, this phenotype was fully explained by increased bone formation, whereas bone resorption was not affected. These findings demonstrated that the inhibitory influence on bone formation is mediated by the CTR in osteoclasts, which led us to study the underlying molecular mechanisms by cell culture experiments.

### CT negatively regulates S1P release from osteoclasts

**Figure 4:**
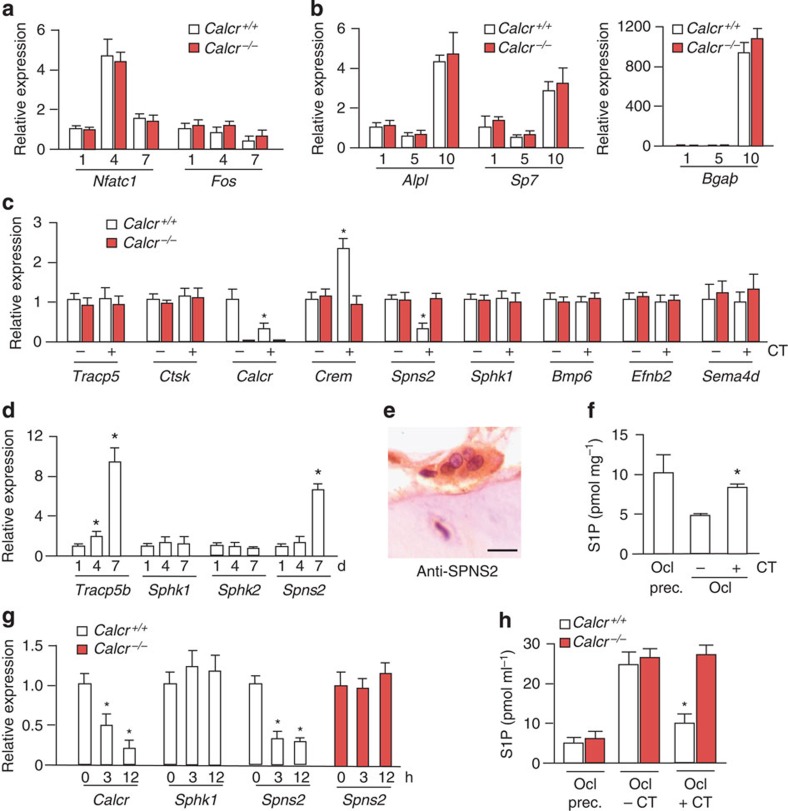
CT inhibits S1P release from osteoclasts. (**a**) qRT–PCR expression analysis for the indicated genes during differentiation of wild-type and *Calcr*^*−/−*^ bone marrow cells into osteoclasts; *n*=3 cultures per group. (**b**) qRT–PCR expression analysis for the indicated genes during differentiation of wild-type and *Calcr*^*−/−*^ bone marrow cells into osteoblasts; *n*=3 cultures per group. (**c**) Affymetrix signal intensities (relative to control) for selected genes in wild-type and *Calcr*^*−/−*^ osteoclasts incubated in the absence or presence of CT; *n*=3 cultures per group. *P<0.05 versus untreated *Calcr*^*+/+*^ osteoclasts. (**d**) qRT–PCR expression analysis for the indicated genes during differentiation of wild-type osteoclasts; *n*=4 cultures per group. *P<0.05 versus day 1. Data are representative of three independent experiments. (**e**) Immunohistochemistry for SPNS2 in an iliac crest biopsy section from a skeletal-intact donor. Scale bar, 20 μm. (**f**) Intracellular S1P concentrations in osteoclast precursors or differentiated wild-type osteoclasts incubated with or without CT for 24 h; *n*=3 cultures per group. *P<0.05 versus untreated osteoclasts. Data are representative of two independent experiments. (**g**) qRT–PCR expression analysis for *Calcr, Sphk1* and *Spns2* in wild-type and *Calcr*^*−/−*^ osteoclasts incubated with CT for the indicated time; *n*=4 cultures per group. *P<0.05 versus control. Data are representative of three independent experiments. (**h**) Extracellular S1P concentrations in conditioned medium of wild-type and *Calcr*^*−/−*^ osteoclast precursors (Ocl prec.) or differentiated osteoclasts incubated with or without CT for 24 h; *n*=3 cultures per group. *P<0.05 versus untreated osteoclasts. Data are representative of three independent experiments. All error bars indicate s.d. *P* values were assessed by two-tailed Student’s *t*-test.

We first confirmed the absence of an endogenous defect of CTR-deficient bone marrow cells in terms of osteoclast differentiation, as suggested by the previous data (Figs 1c and 2e). More specifically, we monitored expression of two key regulators of osteoclastogenesis, Nfatc1 and Fos, during the course of differentiation and found no difference between wild-type and *Calcr*^−/−^ cultures (Fig. 4a). Similar observations were made when the bone marrow cells were stimulated to differentiate into osteoblasts, where we did not observe differential expression of osteogenic markers, such as *Alpl*, *Sp7* and *Bglap* (Fig. 4b). We next isolated RNA from wild-type and *Calcr*^−/−^ osteoclasts treated with CT for 6 h to perform genome-wide expression analysis. This allowed us to identify at least 29 genes regulated by CT specifically in wild-type osteoclasts (Supplementary Fig. 3a). Our analysis revealed that the expression of well-established osteoclast markers, such as *Tracp5* and *Ctsk*, was not affected by CT administration, while the expression of *Calcr* and *Crem,* the two previously known CT target genes in osteoclasts^25^,^26^, was regulated as expected (Fig. 4c). Most importantly, we identified *Spns2* as a gene negatively regulated by CT specifically in wild-type osteoclasts. *Spns2* encodes a transmembrane protein involved in the secretion of S1P^27^,^28^, which has been shown to function as an osteoanabolic factor coupling bone formation to resorption *in vitro*^17^,^18^,^29^. Since this raised the possibility that the CTR-dependent control of bone formation is mediated by reducing S1P release from osteoclasts, and since other genes with a putative role in osteoclast to osteoblast communication (*Sphk1, Bmp6, Efnb2* and *Sema4d*) were not differentially expressed, we focused on *Spns2* as a relevant downstream target of CT in the control of bone formation.

Consistent with the suspected role of *Spns2* in osteoclast to osteoblast communication, we observed that its expression increased during osteoclastogenesis of wild-type cells, in contrast to *Sphk1* and *Sphk2*, the two genes required for intracellular S1P production (Fig. 4d). Likewise, immunohistochemistry on human bone sections revealed that SPNS2 was readily detectable in multinucleated osteoclasts at the bone surface (Fig. 4e). Consistent with the higher expression of *Spns2* in differentiated osteoclasts, we found that intracellular S1P levels declined during the course of osteoclastogenesis and that CT administration significantly increased them (Fig. 4f). Likewise, quantitative PCR with reverse transcription (qRT–PCR) confirmed that *Spns2* expression is negatively regulated by short-term administration of CT to wild-type osteoclasts, but not to *Calcr*^−/−^ osteoclasts (Fig. 4g). As it was previously demonstrated that protein kinase A (PKA), protein kinase C (PKC) and phospholipase C (PLC) are the major intracellular effectors of CTR-dependent signalling^25^,^30^,^31^, we investigated which of these pathways is involved in the transcriptional regulation of *Spns2* by CT. Here we found that the inhibition of *Spns2* expression by CT was abrogated in the presence of the PLC inhibitor U-73122, but not by a PKC or PKA inhibitor, indicating an involvement of the PLC-signalling pathway (Supplementary Fig. 3b). In addition, we found that Spns2 protein levels were markedly decreased after 9 h of CT administration (Supplementary Fig. 3c), thereby supporting our conclusion that Spns2 is a direct target of CT in osteoclasts. To demonstrate the functional consequence of this regulation, we determined S1P levels in the medium of wild-type and *Calcr*^−/−^ osteoclast cultures. In both wild-type and *Calcr*^−/−^ osteoclast cultures extracellular S1P levels increased upon differentiation, whereas CT administration for 24 h resulted in a significant reduction only in wild-type cultures (Fig. 4h). Taken together, these results suggested that the CTR controls bone formation by reducing S1P release from osteoclasts, which led us to address the question as to which of the five known S1P receptors^32^,^33^ is involved in the regulation of bone formation downstream of CT.

### Control of bone formation by S1P_3_

**Figure 5:**
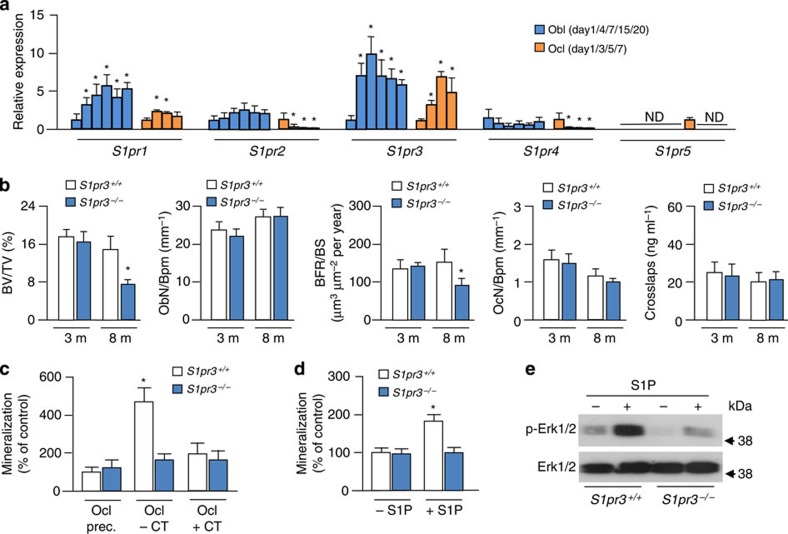
S1P_3_ controls bone formation. (**a**) qRT–PCR expression analysis for the indicated genes in primary osteoblasts (Obl) or osteoclasts (Ocl) at different days of differentiation (ND, non-detectable). Data are representative of two independent experiments. (**b**) Quantification of BV/TV, ObN/Bpm, BFR/BS, OcN/Bpm and measurement of serum crosslaps in 3- and 8-month-old female wild-type and *S1pr3*^−*/*−^ mice; *n*=5 mice per group. *P<0.05 versus wild-type. (**c**) Quantification of mineralization in wild-type and *S1pr3*^−*/*−^ primary osteoblasts differentiated for 10 days in the presence of conditioned medium from wild-type osteoclast precursors (Ocl prec.; control) or differentiated osteoclasts being incubated in the absence or presence of CT; *n*=3 cultures per group. *P<0.05 versus control. Data are representative of two independent experiments. (**d**) Quantification of mineralization in wild-type and *S1pr3*^−*/*−^ primary osteoblasts differentiated for 10 days in the absence or presence of S1P; *n*=3 cultures per group. *P<0.05 versus control. Data are representative of two independent experiments. (**e**) Western Blot monitoring Erk1/2 phosphorylation in wild-type and *S1pr3*^−*/*−^ primary osteoblasts with or without S1P treatment for 15 min (uncropped blots are presented in Supplementary Fig. 5). All error bars indicate s.d. *P* values were assessed by two-tailed Student’s *t*-test.

We next determined the expression of *S1pr1-5* in different tissues as well as in primary bone cells by RT–PCR (Supplementary Fig. 4a) and during the course of their differentiation by qRT–PCR (Fig. 5a). Here we found that *S1pr1* and *S1pr3* were differentially expressed during osteoblastogenesis, both increasing at early stages of differentiation. Since *S1pr1*-deficient mice die *in utero*^34^, in contrast to *S1pr3*-deficient mice, which do not display obvious abnormalities^35^, we first analysed the skeletal phenotype of *S1pr3*-deficient mice. Our histomorphometric analysis revealed that bone remodelling parameters did not differ between wild-type and *S1pr3*-deficient mice at 3 months of age. In contrast, 8-month-old *S1pr3*-deficient mice displayed osteopenia and reduced bone formation, while bone resorption parameters were unaffected (Fig. 5b). We additionally generated mice lacking *S1pr1* specifically in osteoblasts, but here we failed to detect alterations in bone mass or bone remodelling parameters at both ages (Supplementary Fig. 4b–d). These findings identified S1P_3_, encoded by the *S1pr3* gene, as a candidate receptor controlling bone formation in response to S1P, which led us to utilize the *S1pr3*-deficient mouse model for additional experiments.

**Figure 6:**
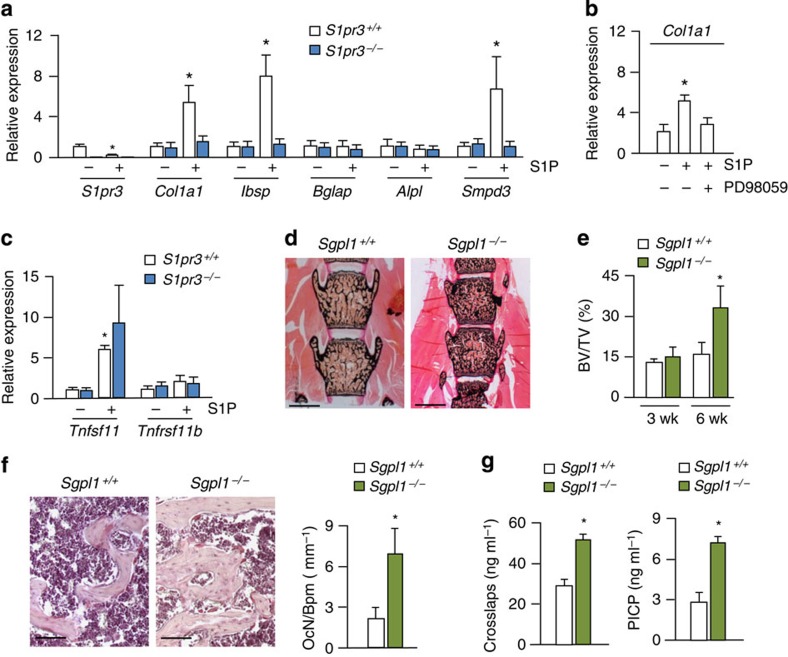
S1P affects gene expression in osteoblasts and accelerates bone turnover. (**a**) qRT–PCR expression analysis for the indicated genes in wild-type and *S1pr3*^−*/*−^ osteoblasts incubated in the absence or presence of S1P for 6 h; *n*=3 cultures per group. *P<0.05 versus control. Data are representative of two independent experiments. (**b**) qRT–PCR expression analysis for *Col1a1* in wild-type osteoblasts stimulated with S1P in the absence or presence of PD98059 as indicted; *n*=3 cultures per group. *P<0.05 versus control. (**c**) qRT–PCR expression analysis for the indicated genes in wild-type and *S1pr3*^−*/*−^ osteoblasts incubated in the absence or presence of S1P for 6 h; *n*=3 cultures per group. *P<0.05 versus control. Data are representative of two independent experiments. (**d**) Von Kossa/van Gieson-staining of non-decalcified spine sections from 6 weeks old wild-type and *Sgpl1*^−*/*−^ mice. Scale bar, 500 μm. (**e**) Quantification of BV/TV in 3 and 6 weeks old female wild-type and *Sgpl1*^−*/*−^ mice; *n*≥3 mice per group. *P<0.05 versus wild-type. (**f**) TRAP activity staining reveals a high number of multinucleated osteoclasts (stained in red) in decalcified spine sections from 6 weeks old *Sgpl1*^−*/*−^ mice. Scale bar, 50 μm. Quantification of OcN/Bpm is shown on the right; *n*=3 mice per group. *P<0.05 versus wild-type. (**g**) Serum crosslaps and PICP concentrations in 6 weeks old female wild-type and *Sgpl1*^−*/*−^ mice; *n*=3 mice per group. *P<0.05 versus wild-type. All error bars indicate s.d. *P* values were assessed by two-tailed Student’s *t*-test.

Here we first cultured primary calvarial osteoblasts from wild-type and *S1pr3*^−/−^ mice in the presence of conditioned medium derived from osteoclast progenitors or from osteoclasts terminally differentiated with or without CT. After 10 days of culture we quantified matrix mineralization and found that only conditioned medium of osteoclasts cultured without CT caused on osteoanabolic effect in wild-type cultures, whereas *S1pr3*^−/−^ cells did not respond (Fig. 5c). Likewise, S1P significantly increased matrix mineralization in wild-type, but not in *S1pr3*^−/−^ bone marrow-derived osteoblast cultures (Fig. 5d). Moreover, S1P treatment resulted in a rapid phosphorylation Erk1/2 in primary calvarial osteoblasts from wild-type, but not from *S1pr3*^−/−^ mice (Fig. 5e, Supplementary Fig. 5). To analyse immediate effects on gene expression we treated wild-type and *S1pr3*^−/−^ bone marrow cells at day 10 of osteogenic differentiation with S1P for 6 h and subsequently performed qRT–PCR for selected genes (Fig. 6a). Similar to the effect of CT on *Calcr* expression in osteoclasts, S1P negatively regulated *S1pr3* expression in wild-type cultures. Importantly, short-term S1P administration caused an S1P_3_-dependent increase in the expression of *Col1a1, Ibsp*, and *Smpd3*, all of which are involved in bone matrix production by osteoblasts^36^,^37^,^38^, and addition of the MEK1 inhibitor PD98059 blunted the stimulatory effect of S1P on *Col1a1* expression (Fig. 6b). Taken together, these results suggested that S1P_3_ is one relevant receptor acting downstream of CT to control bone formation.

Since S1P has been shown to induce expression of the pro-osteoclastogenic cytokine Rankl in osteoblasts, we additionally performed qRT–PCR to assess transcription of the genes encoding Rankl (*Tnfsf11*) or its decoy receptor Opg (*Tnfrsf11b*). While *Tnfsf11* expression was induced by S1P stimulation of wild-type and *S1pr3*^−/−^ bone marrow cells, *Tnfrsf11b* was not affected (Fig. 6c). These results raised the question as to why the suspected increase of S1P release in *Calcr*^−/−^ mice does not amplify osteoclastogenesis. In an attempt to address this question, we took advantage of *Sgpl1*^−/−^ mice, lacking S1P-lyase, an enzyme required for S1P degradation^39^. Due to high extracellular S1P levels, *Sgpl1*^−/−^ mice display various abnormalities and reduced life span (29 days in average). Remarkably, however, their bone mass was progressively increasing and approximately doubled compared with wild-type littermates in all animals reaching the age of 6 weeks (Fig. 6d,e). Of note, TRAP activity staining combined with cellular histomorphometry demonstrated increased osteoclastogenesis in *Sgpl1*^−/−^ mice (Fig. 6f). Importantly, these osteoclasts were functionally active, as the serum crosslaps concentrations were significantly increased, and the same was the case for PICP, a biomarker of bone formation (Fig. 6g). These findings fully confirm the osteoanabolic function of S1P *in vivo* and suggest that an S1P-driven high bone turnover is only detectable when the extracellular S1P level exceed a certain threshold.

### *S1pr3*-deficiency abrogates osteoanabolic influences

**Figure 7:**
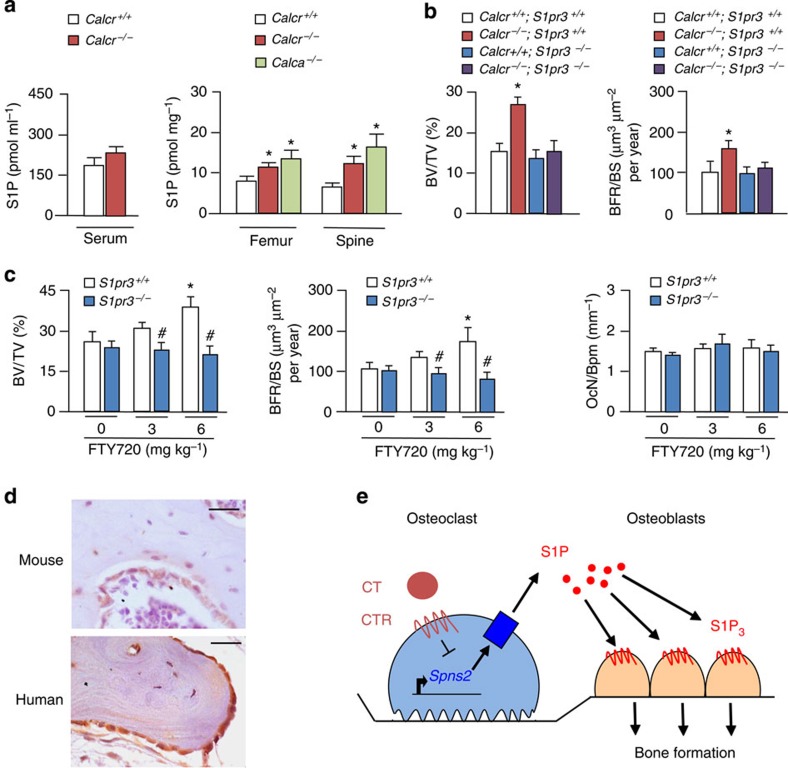
*S1pr3*-deficiency abrogates the osteoanabolic effect caused by CTR deficiency or FTY720. (**a**) S1P levels in serum and bones from 6-month-old female wild-type and *Calcr*^−*/*−^ mice, and in bones from 6-month-old *Calca*^−*/*−^ mice; *n*=5 mice per group. *P<0.05 versus wild-type controls. (**b**) Quantification of BV/TV and BFR/BS in 4-month-old female mice of the indicated genotypes; *n*=5 mice per group. *P<0.05 versus wild-type. (**c**) Quantification of BV/TV, BFR/BS and OcN/Bpm in 4-month-old female mice of the indicated genotypes receiving daily injections of vehicle or FYT720 at two different doses; *n*=6 mice per group. *P<0.05 versus vehicle-treated *S1pr3*^*+/+*^ mice. ^#^*P*<0.05 versus wild-type. (**d**) Immunohistochemistry for S1P_3_ in spine sections from wild-type mice (top) and in iliac crest biopsy sections of an individual with Paget’s disease of bone (bottom). Scale bars, 25 μm (top) and 50 μm (bottom). (**e**) Schematic representation of the proposed function of the CTR in bone formation. All error bars indicate s.d. *P* values were assessed by two-tailed Student’s *t*-test.

To analyse if S1P is responsible for the high bone mass phenotype of *Calcr*^−/−^ mice, we determined S1P levels in serum and bones of 6-month-old wild-type and *Calcr*^−/−^ littermates. Here we did not identify differences in circulating S1P levels, yet S1P concentrations were significantly increased in femur and spine extracts of *Calcr*^−/−^ mice, and the same was the case in *Calca*^−/−^ mice (Fig. 7a). We next generated mice lacking both, the CTR and S1P_3_, and compared their skeletal phenotype to wild-type, *Calcr*^−/−^ and *S1pr3*^−/−^ controls at 4 months of age. Here we observed the expected increase of trabecular bone mass and bone formation in *Calcr*^−/−^ mice, yet this phenotype was not present in *Calcr*^−/−^ mice additionally lacking of S1P_3_ (Fig. 7b). Since it was described that daily injection of the nonselective S1P receptor agonist FTY720 protects against ovariectomy-induced bone loss in wild-type mice^40^, we further investigated whether this effect is attributable to an S1P_3_-dependent increase in bone formation. Therefore, we analysed the skeletal phenotype of wild-type and *S1pr3*^−/−^ mice that received daily vehicle or FTY720 injections for 4 weeks, starting at the age of 3 months. FTY720 administration at a dose of 6 mg kg^−1^ resulted in a significantly increased trabecular bone volume in wild-type mice, whereas *S1pr3*^−/−^ mice did not respond (Fig. 7c). Most importantly, although osteoclast parameters were not different in the six groups of mice, the bone formation rate was significantly increased by FTY720 administration in wild-type, but not *S1pr3*^−/−^ mice.

Since these results suggested that S1P_3_ might be an attractive target for osteoanabolic therapy, we further assessed whether its presence on osteoblasts is also detectable in human bone sections. Using immunohistochemistry we found that S1P_3_ was detectable not only in spine sections from wild-type mice, but also in iliac crest biopsy sections from an individual with Paget’s disease of bone, a condition characterized by a local increase of bone remodelling (Fig. 7d). Taken together, our findings provide a cellular and molecular explanation for the role of CT in mammalian physiology and identify a physiologically relevant coupling mechanism in bone remodelling that can be targeted for osteoanabolic treatment of bone loss disorders (Fig. 7e).

## Discussion

Our results have identified a cellular and molecular explanation for the previously ambiguous role of CT in mammalian physiology. They demonstrate that bone formation is controlled by the CTR in osteoclasts, where CT reduces S1P secretion by inhibiting *Spns2* expression. In turn, the high bone mass phenotype in mice lacking the CTR is the consequence of locally increased S1P levels, which is best underscored by the phenotypic rescue through additional deletion of S1P_3_. These findings are important and clinically relevant for several reasons. First, they demonstrate that the primary physiological function of mammalian CT in bone remodelling is entirely different from its pharmacologic action, similar to PTH, a hormone physiologically activating osteoclastogenesis, but pharmacologically increasing bone formation^41^. Second, they fully support the previously suggested role of S1P as a factor coupling bone formation to bone resorption, which was based on *in vitro*- and *ex vivo*-evidence^17^,^18^,^29^. Third, they have identified two promising target molecules for an osteoanabolic treatment of bone loss disorders, and it is important to state that the CTR and S1P_3_ are both serpentine receptors, representing the major class of target proteins for drug development^32^,^33^,^42^.

Since CT was initially identified as a potent inhibitor of bone resorption at high pharmacological doses, it has long been considered as surprising that thyroidectomy does not cause osteopenia, and that bone mineral density is even decreased in individuals with MTC^12^. During the course of our study we identified three patients with CT deficiency due to thyroiditis, and three patients with high CT serum levels due to MTC (Supplementary Table 4). Dual-energy X-ray absorptiometry (DEXA) revealed high bone mineral density in patients with CT deficiency, whereas the opposite was observed in patients with CT excess (Supplementary Fig. 6a,b). We additionally recruited 17 previously thyroidectomized individuals (Supplementary Table 5) to perform DEXA analyses, where we observed a significant positive correlation between bone mineral density and time after thyroidectomy (Supplementary Fig. 6c). These findings, despite several limitations of our analysis, are principally in agreement with the data obtained in mouse models, and they provide further evidence for a physiological role of mammalian CT as an inhibitor of bone formation. At that point it is again important to state that many conclusions regarding the functions of CT are based on experiments performed with salmon CT. This protein shares only 50% sequence homology to human CT, which may not only translate into different binding characteristics to the CTR, but also in differential effects on bone remodelling. Therefore, our findings do not at all question the anti-resorptive effect of pharmacological salmon CT administration, they only show that mammalian CT has another influence under physiological conditions.

We and others have previously shown that mice lacking CT or one allele of the CTR display increased bone formation^14^,^15^,^19^. Albeit these data already suggested that the primary physiological function of CT is different from its pharmacologic effect, there were still several remaining questions^43^. In fact, while the homozygous deletion of exons 6 and 7 of the *Calcr* gene, which encode the CTR binding site, has been reported to cause embryonic lethality^19^, a *CMV-Cre*-mediated deletion of the 3′-*Calcr* exons 13 and 14 did not result in embryonic lethality or obvious alterations of bone remodelling, which was potentially explained by insufficient recombination^44^. On the basis of these inconsistencies, it was important that we could demonstrate loss of CTR expression and function in our mouse model by several means, and that we could identify increased bone formation as their only phenotype. Although we do not have a sufficient explanation for the previously described lethality of mice lacking the *Calcr* exons 6 and 7, it is important to state that one difference between the two targeting strategies is the absence or presence of the Neo^R^ cassette. In fact, since there are several reports demonstrating a phenotypic influence of such an insertion^45^,^46^,^47^, we have applied Flp-mediated recombination to remove the Neo^R^ cassette before our phenotypic analysis. Our data clearly demonstrated that the major physiological function of the murine CTR is to limit bone formation, which led us to address the remaining questions about the underlying cellular and molecular mechanism.

Since the CTR is known to be present on hypothalamic neurons and differentiated osteoclasts, and since recent evidence has suggested that it might also be expressed by terminally differentiated osteoblasts^48^, we specifically deleted the *Calcr* exons 6 and 7 in the hypothalamus, in osteoblasts or in cells of the osteoclast lineage to uncover the cellular and molecular mechanism explaining the inhibitory effect of CT on bone formation. The subsequent skeletal analysis demonstrated that the presence of the CTR in osteoclasts is required for regulating the activity of osteoblasts, thereby contradicting the hypothesis raised by us and others that CT inhibits bone formation through a hypothalamic relay^10^,^19^,^43^. Instead, our data demonstrate that CT and the CTR are involved in the molecular crosstalk between osteoclasts and osteoblasts. Clinically, there are several bone remodelling disorders where increased bone formation is triggered by excessive osteoclastogenesis, the most evident example being Paget’s disease of bone^49^,^50^,^51^,^52^. Molecularly, several tissue culture experiments together with genetic evidence from mouse models have led to the identification of candidate molecules mediating this function, such as EphrinB2, BMP6, Sema4d or S1P^17^,^18^,^53^,^54^.

In the light of this knowledge, we performed genome-wide expression analysis with the aim to identify a coupling factor-encoding gene being regulated in a CTR-dependent manner. Here we did not observe an immediate transcriptional regulation of theses genes by CT, and the same was the case for *Sphk1*. In the context of S1P this latter finding is particularly important, as differential *Sphk1* expression during osteoclastogenesis was the first experimental evidence for S1P as a relevant coupling factor in bone remodelling^18^. Instead, we observed a CTR-dependent negative regulation of *Spns2* expression, which was potentially important, since this gene encodes a transmembrane protein required for S1P release from various cell types^27^,^28^,^55^. Our qRT–PCR expression analysis revealed that *Spns2* expression increases during osteoclastogenesis, similar to extracellular S1P levels. Likewise, short-term administration of CT to wild-type, but not to *Calcr*^−/−^ osteoclasts, caused a significant reduction of both, *Spns2* expression and extracellular S1P accumulation. Consistent with these *in vitro* experiments, *Calcr*^−/−^ mice displayed increased S1P levels in bone extracts, and their skeletal phenotype was normalized by the additional absence of S1P_3_. Taken together, these results demonstrate that the indirect action of CT on bone formation is explained by locally increased S1P concentrations, thereby supporting the concept that S1P receptors serve an osteoanabolic function.

Although we cannot fully rule out a contribution of additional S1P receptors, our results demonstrate that S1P_3_ is one relevant receptor required to promote bone formation in response to S1P. While the lack of a bone remodelling phenotype in 3-month-old *S1pr3*-deficient mice is potentially explained by functional redundancy, most likely involving *S1pr1* based on its differential expression in osteoblasts, it was evident that *S1pr3*-deficiency blunted the effects of S1P *in vitro,* but also *in vivo* in the context of CTR deficiency. From a therapeutic perspective, the finding that FTY720 causes an S1P_3_-dependent osteoanabolic effect is of particular interest, as this nonselective S1P receptor agonist has already been approved for the treatment of individuals with multiple sclerosis^56^,^57^. In a previous study, FTY720 administration (3 mg kg^−1^) was found to protect against ovariectomy-induced bone loss due to a negative effect on osteoclast attachment to the bone matrix^40^. Although we principally used the same strategy of FTY720 administration, it is important to clarify the three relevant modifications compared with this former experiment. First, since we only observed a trend, and not a significant increase of the trabecular bone volume by daily injection of FTY720 at a dose of 3 mg kg^−1^, we repeated the experiment with a higher dose (6 mg kg^−1^) to demonstrate an osteoanabolic effect in wild-type mice. Second, our analysis included dynamic histomorphometry, which enabled us to identify differential effects on bone formation caused by FTY720 administration. And third, since we applied the same strategy to *S1pr3*-deficient mice, we were able to show that the osteoanabolic effect of FTY720 administration is mediated through S1P_3_.

Although these results provide an important proof of principle for the therapeutic potential of S1P receptor stimulation in osteoporotic individuals, they do not directly translate into clinical applicability, since the dosage used for treatment of multiple sclerosis is much lower (up to 5 mg per day) as compared with the animal experiments. Importantly, however, while it is unlikely that FTY720 itself could be applied for osteoanabolic treatment in humans, it might be a promising approach to develop selective S1P_3_ agonists and to analyse their potential for osteoporosis therapy. The osteoanabolic potential of S1P and its remarkable influence on bone remodelling is probably best underscored by the phenotype of *Sgpl1*^−/−^ mice. These mice have been described to display high extracellular S1P levels causing various abnormalities and early postnatal lethality^39^. Moreover, a non-quantitative analysis of their skeletal phenotype demonstrated high bone mass, which was interpreted as osteopetrosis, that is, osteoclast dysfunction. Our histomorphometric analysis confirmed that the trabecular bone mass is dramatically increased in all *Sgpl1*-deficient mice reaching the age of 6 weeks (approximately 15%). Importantly, however, the number of functionally active osteoclasts was significantly increased towards wild-type littermates, and the same was the case for serum PICP, indicating excessive bone formation. In conclusion, *Sgpl1*-deficient mice display high bone turnover triggering a progressive bone mass increase, which occurs despite the existence of various organ abnormalities. These findings are in full agreement with the suspected key role of S1P in bone remodelling and its ability to act as an osteoanabolic molecule.

Therefore, our collective findings do not only expand our current understanding of bone remodelling, they also identify two serpentine receptors specifically regulating bone formation. Since serpentine receptors represent the major class of target proteins for currently available drugs, it is conceivable to speculate that the development of CTR-specific antagonists and/or S1pr3-specific agonists might be a promising approach to counteract bone loss in osteoporosis or other skeletal disorders.

## Methods

### Mouse models

To obtain *Calcr*^*fl/fl*^ mice a targeting construct was generated, where we placed a Frt-neomycin-Frt-loxP cassette (kindly provided by G. Schuetz, Heidelberg, Germany) 5′ of exon 6 and a single loxP site 3′of exon 7, the latter one being inserted by ET recombination according to standard protocols. The construct was electroporated into embryonic stem cells R1, which were then screened for homologous recombination by Southern blotting following DNA digestion by Hind III. After removal of the neomycin resistance gene by electroporating a Flp-recombinase expression plasmid (kindly provided by G. Schuetz, Heidelberg, Germany), the targeted embryonic stem cells were injected in C57Bl/6J blastocysts to generate chimeric mice, which were then crossed with C57Bl/6 mice to obtain offspring with one floxed *Calcr* allele. These mice were either crossed to obtain *Calcr*^*fl/fl*^ mice or with *CMV-Cre* transgenic mice, resulting in an ubiquitous recombination within the *Calcr* allele, as determined by Southern blotting or PCR genotyping using two primers flanking the loxP site between exons 7 and 8 (5′-TCCTGGGCTGCTGAGAAAGTATC-3′ and 5′-ATGTGATTGGCTGGGCACTG-3′) and one primer located 5′of the loxP site between exons 5 and 6 (5′-AAGACAGATGGTGAGGGCTGACTG-3′). The generation and genotyping of *Calca*^−/−^, αCGRP^−/−^, *S1pr1*^fl/fl^, *S1pr3*^−/−^ and *Sgpl1*^−/−^ mice has been described previously^14^,^34^,^35^,^39^,^58^. The same applies for mice expressing the Cre recombinase under the control of different promoters^22^,^23^,^24^. For the confirmation of cell type-specific *S1pr1* inactivation, a combination of primers (5′-GAGCGGAGGAAGTTAAAAGTG-3′, 5′-CCTCCTAAGAGATTGCAGCAA-3′ and 5′-GATCCTAAGGCAATGTCCTAGAATGGGACA-3′) amplifying either the floxed or the recombined allele. To rule out a possible influence of genetic background, all *in vivo* analyses were performed with littermate controls. Although our initial analysis of the *Calcr*^−/−^ phenotype was done with 12-week-old male and female mice, we used female mice for subsequent analyses with ages ranging from 3 weeks until 18 months, as indicated in the respective figure legends. Animal care and experimental procedures were performed with approval from the animal care committees of the University Medical Center Hamburg-Eppendorf.

### Immunological protein detection

Immunohistochemistry was performed according to standard protocols using polyclonal antibodies against the rat CTR (Acris), human SPNS2 (Lifespan), human S1P_3_ (Lifespan) or mouse S1P_3_ (Santa Cruz), all diluted 100-fold. Human bone sections were derived from decalcified iliac crest biopsies from an individual with M. Paget^52^, or from an autopsy case of a skeletal-intact donor. These studies were approved by the ethics committee of the Aerztekammer Hamburg. For immunohistochemistry the rehydrated sections were pretreated with 0.1% trypsin for 30 min, and then incubated in 3% hydrogen peroxide for 15 min to block endogenous peroxidase activity. Incubation with 5% BSA for 30 min was subsequently performed to block nonspecific binding. Immunohistochemical staining with the primary antibodies was performed overnight at 4 °C. Detection was achieved with a biotinylated secondary goat anti-rabbit IgG (1:200, Dako Cytomation) followed by incubation with a streptavidin/HRP (1:200, Dako Cytomation). Peroxidase activity was detected using DAB (3,3′-diaminobenzidine tetrahydrochloride) as chromogenic substrate (Dako Cytomation). Sections were counterstained with hematoxylin, dehydrated and mounted. For western blotting, whole cells were lysed in RIPA buffer (1% NP-40, 1% sodium desoxycholate, 0.1% sodium dodecylsulfate, 150 mM sodium chloride, 2 mM EDTA and 10 mM sodium phosphate) or in CHAPS extraction buffer (50 mM Tris/Hcl pH 8.0, 150 mM NaCl, 5% glycerol, 1% CHAPS, 2 mM EGTA, 1 mM DTT) containing a protease and phosphatase inhibitor cocktail (Roche). Equal amounts of protein were separated on 12.5% SDS–polyacrylamide gel electrophoresis and transferred to PVDF membranes (Hybond; GE Healthcare). After blocking with Tris-buffered saline containing 0.1% Tween 20 and 5% nonfat dry milk, membranes were incubated overnight at 4 °C with primary antibodies at a dilution of 1:1,000. The antibodies were directed against Spns2 (#ARP56057; Avia Systems Biology), β-actin (#MAB1501; Millipore), phospho-Erk1/2 (#9101; Cell Signaling Technology), or Erk1/2 (#4695; Cell Signaling Technology). Secondary HRP-conjugated antibodies (Dako) were used at a dilution of 1:5,000.

### Cell culture

Primary osteoclasts were generated as described previously^59^. In brief, osteoclast precursor cells were isolated from the bone marrow of 12-week-old mice and differentiated for 4 days in α-MEM containing 10 nM 1,25(OH)_2_ Vitamin D_3_. For the next 6 days, they additionally received M-CSF (20 ng ml^−1^) and RANKL (40 ng ml^−1^) to allow terminal differentiation. The conditioned medium was collected from day 1–4 of culture (osteoclast precursor cells) or from day 7–10 of culture (mature osteoclasts). Primary osteoblasts were isolated by sequential collagenase digestion from the calvariae of 5-day-old mice and differentiated for 10 days in the presence of 25 μg ml^−1^ ascorbic acid and 5 mM β-glycerophosphate^59^. In some cases the culture medium contained 50% of conditioned medium from osteoclast precursor cells or mature osteoclasts (cultured either in the absence or in the presence of 10^*−*7^ M CT for the last 4 days) as indicated. To monitor effects of S1P on osteoblasts, bone marrow cells were differentiated for 10 days in the presence of 25 μg ml^−1^ ascorbic acid and 5 mM β-glycerophosphate with or without S1P (1 μM). For short-term treatment, cells were serum-starved overnight and then stimulated with S1P (1 μM) for 6 h.

### Cellular assays

For binding assays mature osteoclasts were incubated for 2 h with 10^*−*11^ M ^125^I-salmon CT in the absence or presence of 10^*−*8^ M salmon CT. Bound radioactivity was released by 0.5 M NaOH and quantified in a γ-counter. For resorption assays, bone marrow cells were plated on dentin slices and differentiated as described above (with CT added during the last 4 days). After staining with toluidine blue, the resorbed area was quantified using ImageTool software. TRAP activity staining was performed as described previously^59^. In brief, after removal of the medium and two washing steps with phosphate-buffered saline (PBS), cells were fixed with cold methanol for 5 min. After washing and drying, they were stained with Naphthol AS-MX-Phosphate (Sigma) for 30 min before the number of TRAP-positive multinuclear cells per well was determined. For pathway analyses osteoclasts were pre-incubated with inhibitors of PLC (U-73122, Sigma, #U6756; 10 μM), PKC (Chelerythrine chloride, Sigma, #C2932; 10 μM) or PKA (H-89, Sigma, #B1427; 300 nM) for 2 h, followed by the stimulation with 10^*−*7^ M CT for 6 h. Likewise, osteoblasts were pretreated with an Erk inhibitor (PD98059, Cell Signaling Technology #9900, 50 μM) for 2 h before stimulation with 10^*−*6^ M S1P for 6 h. For the assessment of matrix mineralization in primary osteoblast cultures, cells were stained after von Kossa, and the mineralized area was quantified using ImageTool software. For alizarin red staining, cells were incubated with 40 mM alizarin red staining solution (pH 4.2) for 10 min at room temperature after fixation in 90% ethanol. To quantify alizarin red incorporation cells were washed with PBS and fixed in 90% ethanol for 1 h. After washing twice with distilled water, cells were stained with alizarin red S solution (40 mM, pH 4.2) for 10 min. Following additional washing steps with distilled water, the cell-bound alizarin red was dissolved in 10% acetic acid. After incubation for 30 min at room temperature and 10 min at 85 °C, the supernatant of a subsequent centrifugation step was neutralized with 10% ammonium hydroxide solution and the absorbance was measured at 405 nm.

### Biochemical assays

Analysis of mineral homeostasis, kidney function and determination of metabolic and hepatic parameters was carried out in the German Mouse Clinic as described^60^. All parameters were determined with a Beckman-Coulter AU 480 autoanalyzer, with the excecption of glycerol, which was measured using a kit from Randox Laboratories (Krefeld, Germany). Enzyme-linked immunosorbent assay was used to measure concentrations of collagen degradation products (Crosslaps, IDS), osteocalcin (Biomedical Technologies) and PICP (USCN Life Science). S1P concentrations were determined following HPLC analysis with all samples being collected in silicone-coated vessels^61^. The samples were transfered into siliconized glass tubes, internal standard was added and lipids were extracted by the addition of chloroform and methanol/HCl (99.8:0.2 v/v) after prior alkalization by 3 N NaOH. The mixture was centrifuged (300 *g*, 5 min) to obtain the alkaline aqueous phase containing S1P. The organic phase was re-extracted with methanol, 1 N NaCl and 3 N NaOH. The combined aqueous phases were acidified by adding concentrated HCl and extracted twice with chloroform. The resulting organic phases were combined and evaporated by a vacuum system. The dried lipids were redissolved in methanol. Sample analyses were performed by rapid-resolution liquid chromatography–tandem mass spectrometry using an Agilent 1200 Series binary pump, a degasser and an autosampler (Agilent Technologies, Böblingen, Germany). A quadrupole time-of-flight 6530 mass spectrometer (QTOF) equipped with Jet-Stream technology operating in the positive electrospray ionization mode was used for detection (Agilent Technologies). High-purity nitrogen for the mass spectrometer was produced by a nitrogen generator (Parker Balston, Maidstone, UK). Chromatographic separations were obtained using a Waters X-Bridge C18 separation column (4.6 mm × 150 mm, 3.5 μm particle size, 138 Å pore size) with a Waters X-Bridge C18 guard column (4.6 × 20 mm; Waters). We chose a binary solvent system with gradient elution consisting of eluent A (water/formic acid; 100:0.1 v/v); and eluent B (acetonitril/tetrahydrofuran/formic acid; 50:50:0.1 v/v); and a flow rate of 0.5 ml min^−1^ over 15 min. Mass spectrometric measurements were conducted using the following ion source conditions and gas settings for positive liquid chromatography–tandem mass spectrometry: sheath gas temperature=300 °C; sheath gas flow=9 l min^−1^; nebulizer pressure=30 p.s.i.g.; drying gas temperature=300 °C; drying gas flow=8 l min^−1^; capillary voltage=5,400 V; fragmentor voltage=200 V; nozzle voltage=2,000 V; and collision energy=15 V. Quantification was performed using MassHunter Software (Agilent Technologies). A calibration curve of analyte concentration was performed from 1–150 pmol and constructed by linear fitting using the least squares linear regression calculation. The resulting slope of the calibration curve was then used to calculate the concentration of the respective analyte in the unknowns.

### Skeletal analysis

Before their skeletal analysis all mice were injected twice with calcein (30 mg kg^−1^ body weight) 9 and 2 days before sacrifice, which allowed a quantification of the bone formation rate by dynamic histomorphometry^62^. The dissected skeletons were fixed overnight in 4% formalin and then transformed into 80% ethanol, before an initial analysis by contact Xray was performed using a Faxitron Xray cabinet. The μCT scanning was performed using a Scanco μCT 40 applying 55 kV, 145 μA and 200 ms integration time. Cortical bone was delineated and evaluated automatically using the image analysis algorithm provided by the manufacturer. The specific region for analysis of cortical thickness was a 1-mm thick section of the central diaphysis. Bone mineral density in patients, after obtaining informed consent, were measured in spine and both femora using DEXA (GE Lunar Prodigy DF+14622). In a subset of individuals structural bone parameters were determined in the left radius using high-resolution peripheral quantitative computed tomography (Scanco Medical xtremeCT). All blood parameters were determined by the Department of Clinical Chemistry. Exclusion criteria for the patient study were clinically apparent hypo- or hyperparathyroidism, Paget’s disease of bone, impaired renal function, malignancies and metastatic bone disease, and bisphosphonate or teriparatide treatment.

### Histology

Non-skeletal organs were histologically analysed in the German Mouse Clinic as described^60^. All organs were fixed in 4% buffered formalin before being embedded into paraffin. Sections of 2 μm thickness were stained with haematoxylin and eosin according to standard protocols. For bone-specific analyses, dissected skeletons were fixed in 3.7% PBS-buffered formaldehyde for 18 h and then transferred into 80% ethanol. Vertebral bodies L1 to L4 were dehydrated in ascending alcohol concentrations, before they were embedded into methylmethacrylate. Sections of 4 μm thickness were cut in the sagittal plane using a Microtec rotation microtome. These were stained by von Kossa/van Gieson (for static hisotmorphometry) and toluidine blue (for cellular histomorphometry) staining procedures as described^62^. To determine the bone formation rate, all mice were injected twice with calcein 9 and 2 days before being killed. Static, cellular and dynamic histomorphometry at trabecular bone surfaces was carried out according to the guidelines of the American Society for Bone and Mineral Research^63^ using an OsteoMeasure system (Osteometrics Inc., USA). TRAP activity staining was performed on decalcified sections using Naphthol AS-MX phosphate (Sigma) and Fast Red Violet LB salt (Sigma) in 40 mM acetate buffer (pH 5).

### Mice treatment

For induction of calvarial porosity, anaesthetized mice were injected with 10 mg kg^−1^ LPS (*Escherichia coli* O111:B4) onto their calvariae subcutaneously. After 6 days mice were killed and calvariae subjected to μCT analyses as described above. For quantification of the porosity the Axiovision software (Carl Zeiss) was used, while osteoclasts number was determined after TRAP activity staining as described above. Treatment of wild-type and *S1pr3*-deficient mice with FTY720 was performed in accordance to previous study^40^. More specifically 3-month-old mice received daily intraperitoneal injections of FTY720 (3 mg kg^−1^ or 6 mg kg^−1^) or vehicle (PBS containing 5% acidified DMSO and 30% BSA). After 4 weeks of treatment, the mice were analysed for their skeletal phenotype as described above.

### Expression analysis

RNA was isolated using the RNeasyMini kit (Qiagen). For genome-wide expression analysis, 5 μg of RNA were used for first-strand cDNA synthesis. For Gene Chip hybridization, the fragmented cRNA was incubated in hybridization solution at 45 °C for 16 h, before the Gene Chips (Affymetrix MG 430 2.0) were washed using the Affymetrix Fluidics Station 450. Microarrays were scanned with the Affymetrix Gene Chip Scanner 7G, and the signals were processed using GCOS(Affymetrix). The gene expression data have been deposited in GEO under accession code GSE60761. For RT–PCR expression analysis, 1 μg of RNA was reversed transcribed using SuperScriptIII (Invitrogen) according to manufacturer’s instructions. Gene-specific primers were used to amplify fragments of *S1pr1* (5′-GGCTTATCTGGTCCCTCCTC-3′and 5′-CTTTGGCCTCAGCAAATAGC-3′), *S1pr2* (5′-CCCCCTTCCATAAACAACCT-3′and 5′-CCCATACTGCCTCACCTGAT-3′), *S1pr3* (5′-TCTGCTTTCACACAACAGCC-3′and 5′-GCAAGTAGCCAAGGTTGCTC-3′), *S1pr4* (5′-GCAGAAGTCTCCACGTCCTC-3′and 5′-GCTGAGTGACCGAGAAGTCC-3′), *S1pr5* (5′-TATGGCTGCAGCAGAAATTG-3′and 5′-TTCCTCTGTAGCCAGCCACT-3′), and *Gapdh* (5′-GACATCAAGAAGGTGGTGAAGCAG-3′and 5′-CTCCTGTTATTATGGGGGTCTGG-3′), respectively. For qRT–PCR expression analysis, 1 μg of RNA was reversed transcribed using SuperScriptIII (Invitrogen) according to manufacturer’s instructions. qRT–PCR analysis (with *Gapdh* as internal control) was performed using predesigned TaqMan gene expression assays (Applied Biosystems), with the exception of *S1pr2*, where qRT–PCR was performed using SYBR green (5′-CGCAGGCTGGAGCTTAGGGC-3′ and 5′-GCAGGAGCTGGGTGAGCTGC-3′).

### Statistical analysis

The number of samples was chosen according to established standards providing sufficient statistical power to enable the detection of biologically relevant differences in animal models. Mouse data were analysed by two-tailed Student’s *t*-test using Excel software. All data are reported as mean±s.d. Human data were analysed using IBM SPSS Statistics 20. *P*<0.05 was considered statistically significant.

## Authors contributions

T.S. and M.A. designed the research. J.K., A.K.H., A.J., T.K., M.H., I.H-B. designed and performed experiments, P.C-L., A.L., M.K., J.A., J.S., S.S., H.D., T.H., M.N., T.S., S.B., F.B., B.R., E.W., J.C-W, F.N., V.G-D, H.F. M.HdA, E.T., L.C.H, B.K. analysed the data. B.L., S.K., J.C. contributed the reagents and analytic tools.

## Additional information

**Accession codes:** The genome-wide expression data set has been deposited in NCBI Gene Expression Omnibus under accession code GSE60761.

**How to cite this article:** Keller, J. *et al.* Calcitonin controls bone formation by inhibiting the release of sphingosine 1-phosphate from osteoclasts. *Nat. Commun.* 6:5215 doi: 10.1038/ncomms6215 (2014).

## Supplementary Material

Supplementary Information Supplementary Figures 1-6, Supplementary Tables 1-5.
