# Supplementary material for: Calcitonin controls bone formation by inhibiting the release of sphingosine 1-phosphate from osteoclasts
Source: Nat Commun. 2014 Oct 21;5:5215. doi: 10.1038/ncomms6215 (PMC4205484; doi:10.1038/ncomms6215)
Supplement: Supplementary Information — Supplementary Figures 1-6, Supplementary Tables 1-5. [file ncomms6215-s1.pdf]

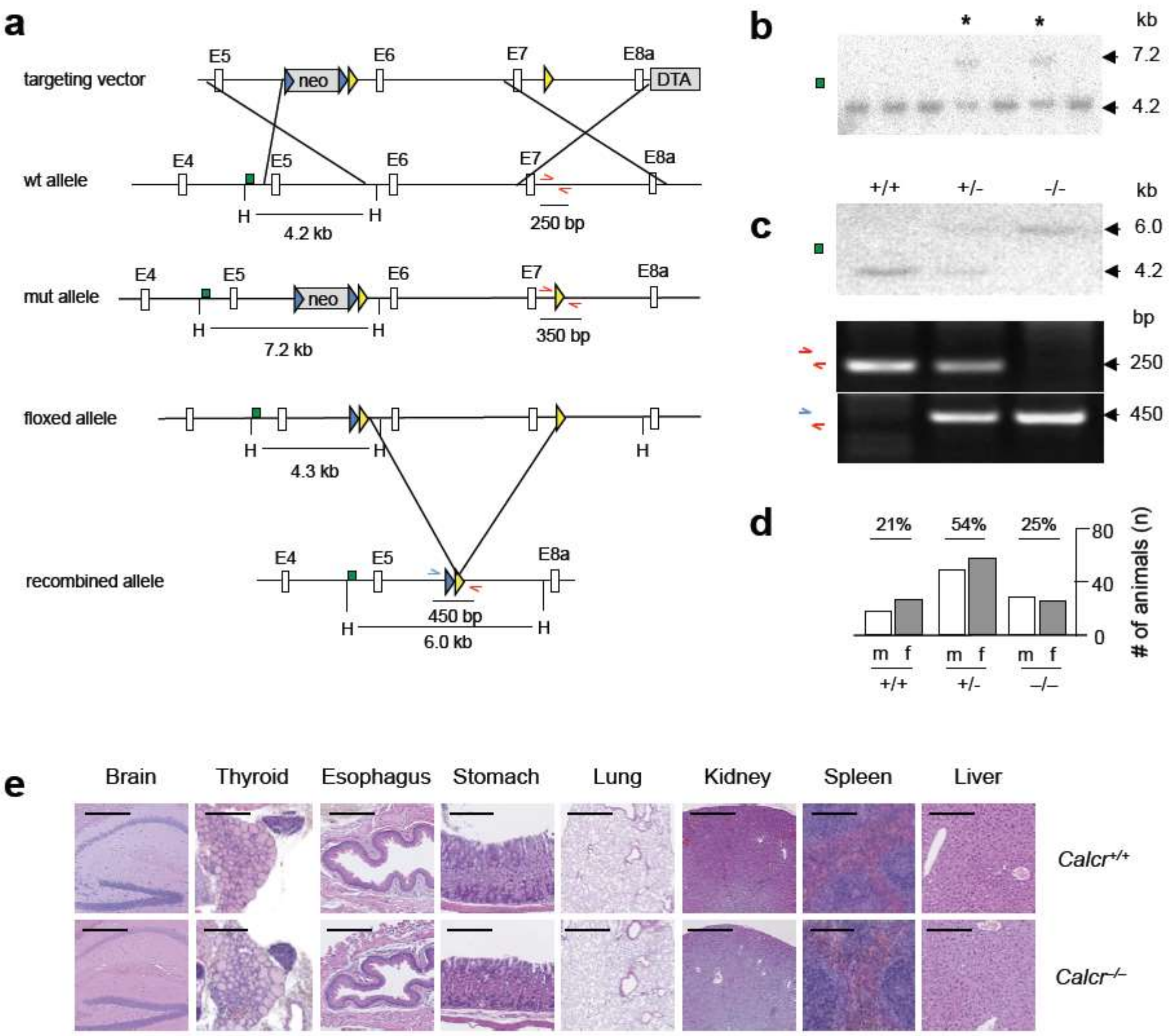

Supplementary Figure 1 | Generation and phenotyping of *Calcr*$^{-/-}$ mice. (**a**) Embryonic stem cells were transfected with a targeting construct carrying a Frt(blue triangles)-Neo^{R}-Frt-loxP (yellow triangles) cassette 5´of the *Calcr* exon 5 and a single loxP-site 3´of the *Calcr* exon 7. After successful homologous recombination (mutant allele) the neomycin resistance was removed by Flp-mediated recombination, resulting in a floxed *Calcr* allele. Following the generation of mice carrying this allele, they were crossed with *CMV-Cre* mice to obtain *Calcr*$^{+/-}$ animals with a recombined allele in all cell types. The probe used for Southern blotting after Hind III (H) digestion is indicated in green, while the primers used for PCR genotyping are indicated as blue and red arrows. (**b**) Southern Blot hybridization showing two embryonic stem cell clones (*) with a mutant allele (additional 7.2 kb fragment). (**c**) Genotyping of the offspring from *Calcr*$^{+/-}$ matings by Southern blotting (top) and PCR (bottom) with the indicated primer pairs. (**d**) Number of male and female mice of the indicated genotypes in the offspring from *Calcr*$^{+/-}$ matings. (**e**) Hematoxylin/eosin-stained sections of the indicated tissues from WT and *Calcr*$^{-/-}$ mice. Scale bars, 200 µm (esophagus, stomach, spleen, liver), 500 µm (brain, thyroid) or 1 mm (lung, kidney).

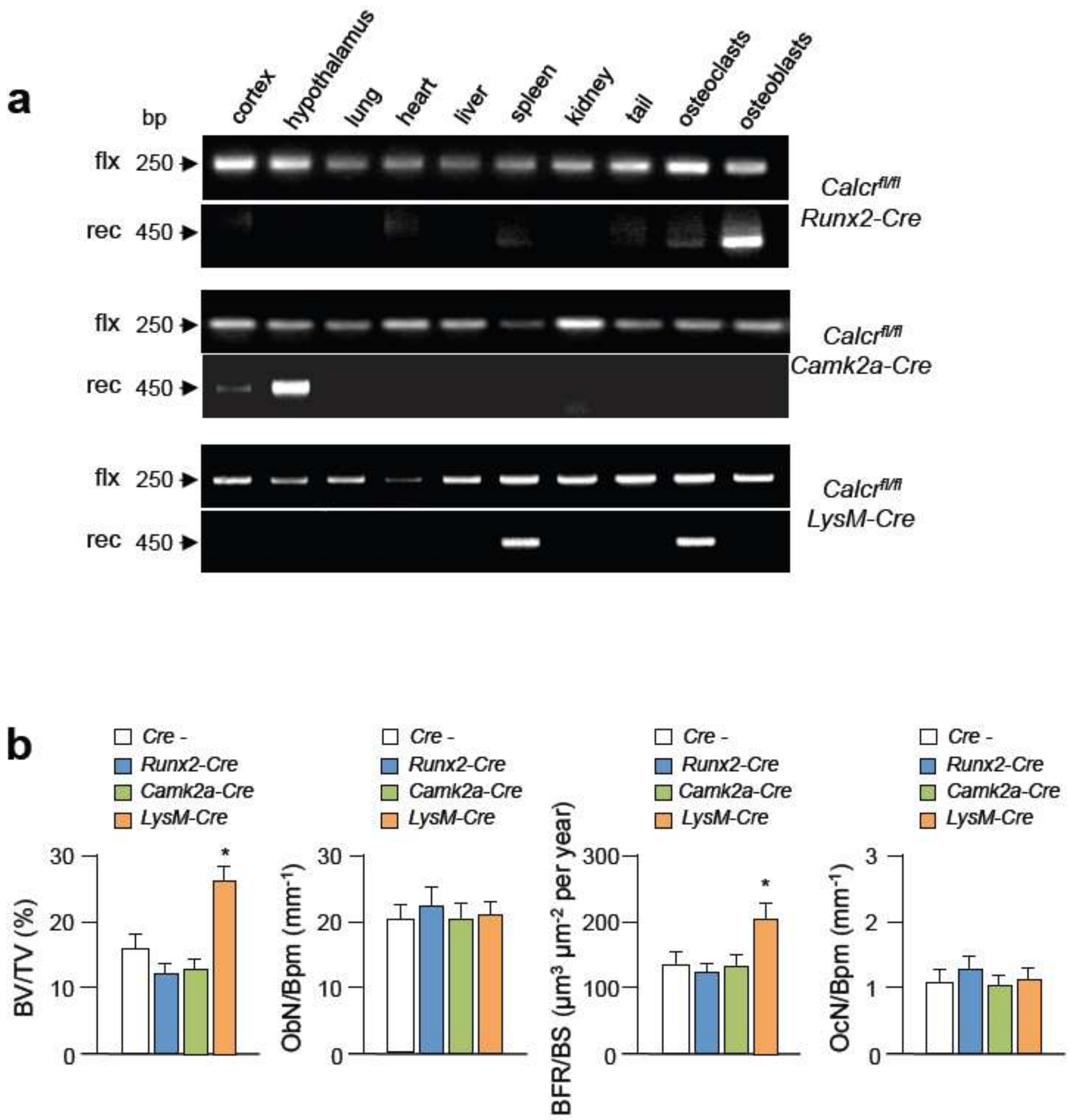

Supplementary Figure 2 | Specific inactivation of the CTR using *Runx2*-, *Camk2a*-, and *LysM*-Cre transgenic mice. (**a**) Genomic PCR for the floxed or recombined *Calcr* allele using DNA from various tissues, as well as primary osteoclasts and osteoblasts at day 7 or 10 of differentiation, respectively. (**b**) Quantification of BV/TV, ObN/Bpm, BFR/BS and OcN/Bpm in 6 months old female mice of the indicated genotypes. n=6. *$p<0.05$ vs *Calcr*$^{fl/fl}$. All error bars indicate s.d. P-values were assessed by two-tailed Student´s *t*-test.

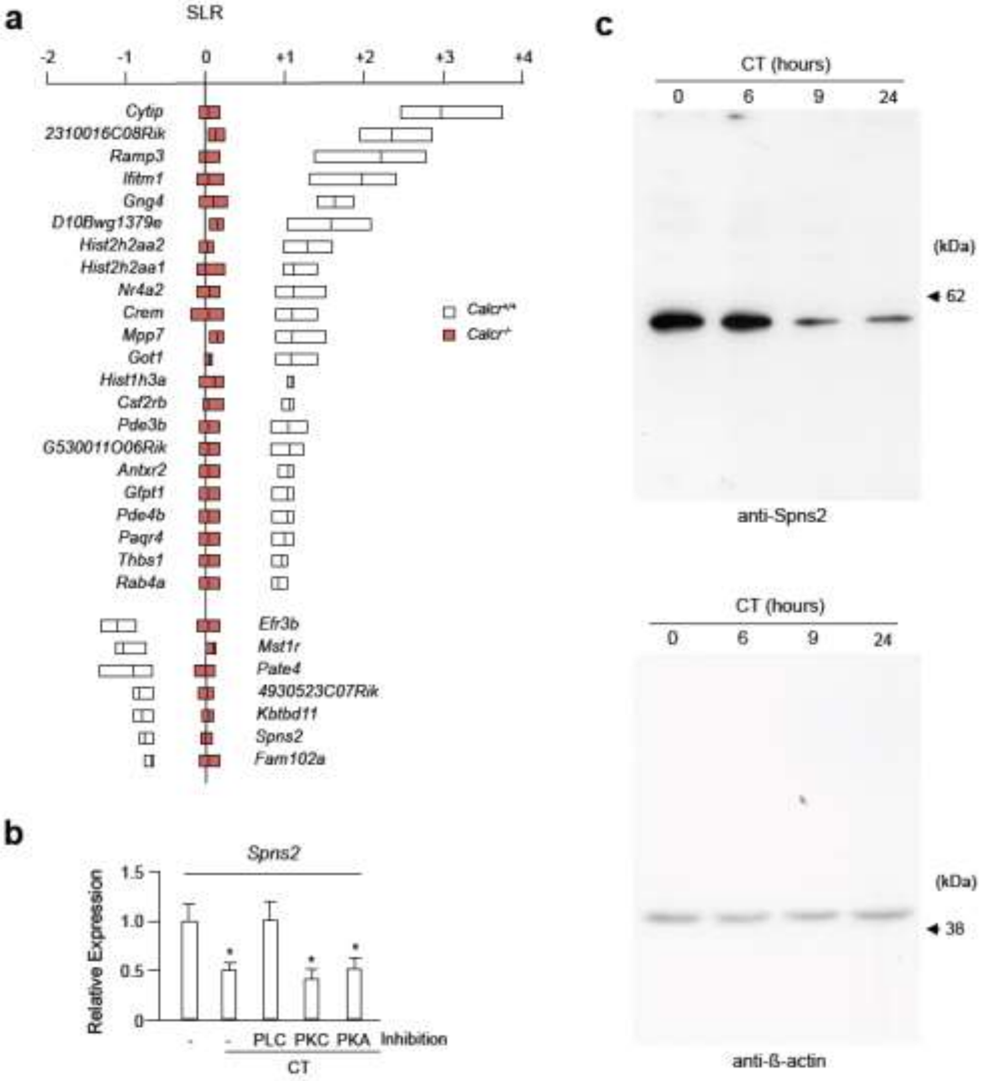

Supplementary Figure 3 | CT-regulated genes in osteoclasts. (a) Osteoclasts from wildtype and *Calcr*$^{-/-}$ mice were incubated with mouse CT for 6 hours before RNA was isolated and subjected to Affymetrix Gene Chip hybridization (n=3 samples from independently isolated cultures). CT-regulated genes were identified based on the finding that the logarithmic ratio of signal intensities (SLR, signal log ratio) between untreated and CT-treated samples was higher than 0.5 (positive regulation) or lower than -0.5 (negative regulation) in all three comparisons of WT cells. The graph shows the SLR range (with the mean SLR indicated by the line) for all CT-regulated genes in wildtype (white bars) and *Calcr*$^{-/-}$ osteoclasts (red bars). (**b**) qRT-PCR expression analysis for *Spns2* in osteoclasts incubated with CT in the presence of inhibitors for specific signaling pathways as indicated. n=4 cultures per group. *$p<0.05$ versus non-treated controls. Error bars indicate s.d. P-values were assessed by two-tailed Student´s *t*-test. Data are representative of two independent experiments. (**c**) Western Blot for Spns2 and ß-actin using CHAPS-lysates of osteoclasts treated with CT for the indicated times.

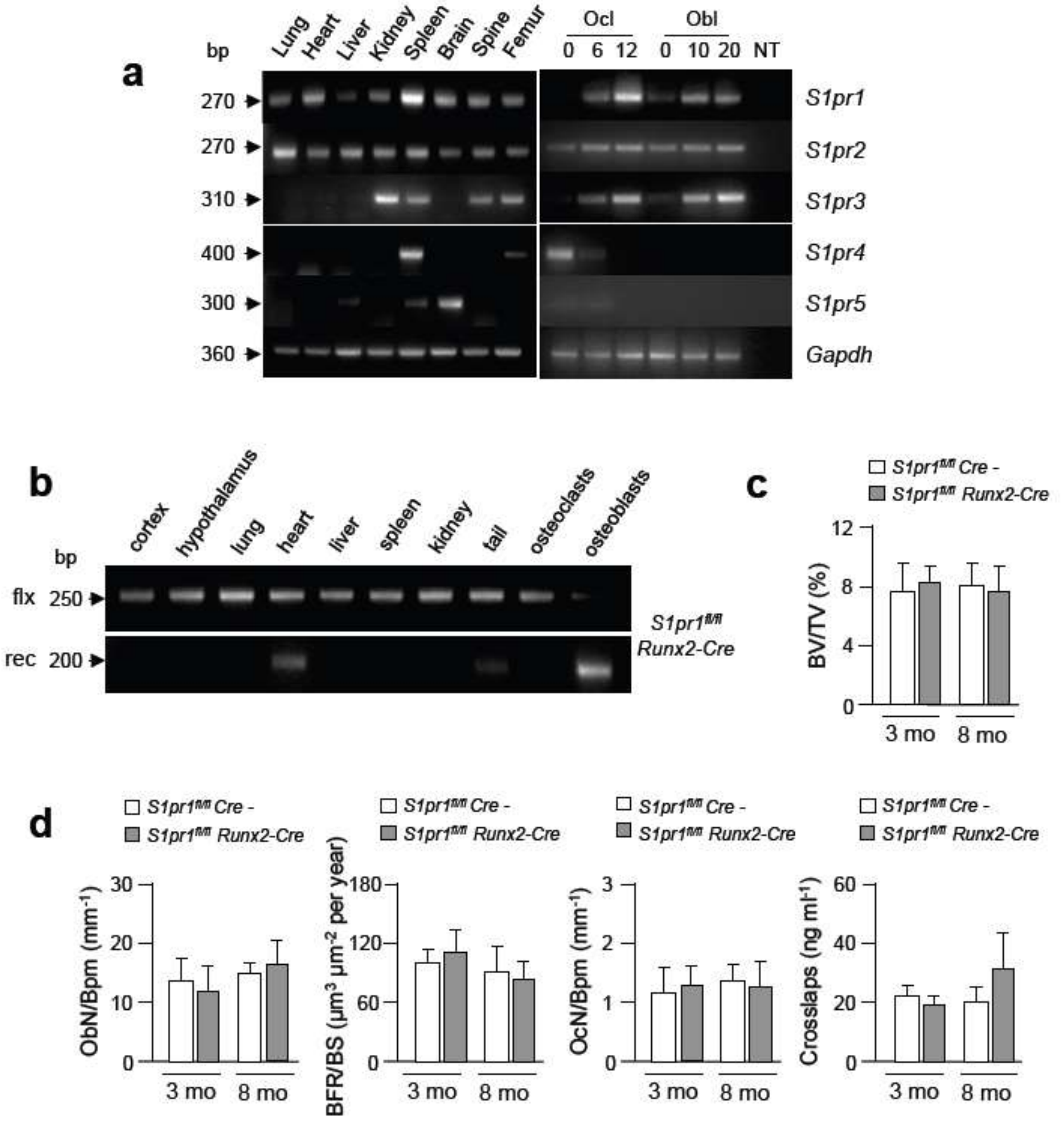

Supplementary Figure 4 | *S1pr1* inactivation in osteoblasts. (**a**) RT-PCR expression analysis for *S1pr* genes in various tissues, as well as in primary osteoclasts (Ocl) or osteoblasts (Obl) at different days of differentiation (NT=no template control). (**b**) Genomic PCR for the floxed or recombined *S1pr1* allele using DNA from various tissues, as well as primary osteoclasts and osteoblasts at day 7 or 10 of differentiation, respectively. (**c**) Quantification of the trabecular bone volume per tissue volume (BV/TV) in 3 and 8 months old female mice of the indicated genotypes. n=5. (**d**) Quantification of the osteoblast number per bone perimeter (ObN/Bpm), the bone formation rate per bone surface (BFR/BS), the osteoclast number per bone perimeter (OcN/Bpm) and measurement of serum crosslaps in the same mice. n=5. All error bars indicate s.d. P-values were assessed by two-tailed Student´s *t*-test.

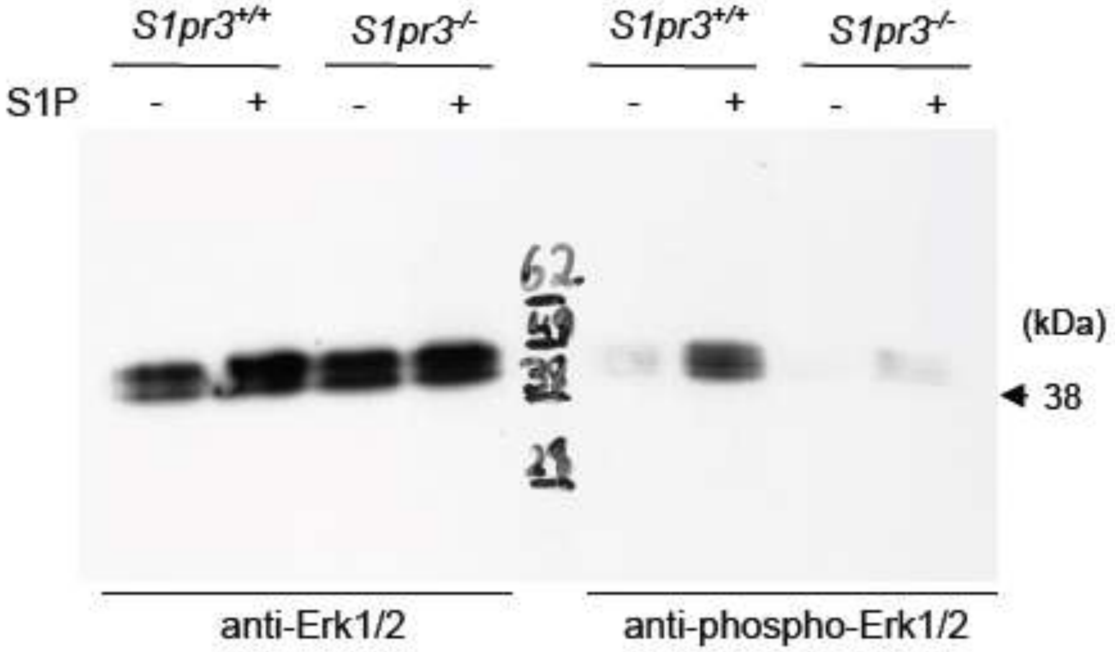

Supplementary Figure 5 | Full scans of Western blots presented in Fig. 5e.

a

	Patient	Total	Spine	Femur
Thyro-iditis	A (f)	+4.8	+4.5	+3.8
	B (f)	+2.8	+3.3	+1.5
	C (f)	+4.3	+5.1	+2.4
MTC	D (f)	-1.9	-1.8	-1.5
	E (m)	-2.0	-1.4	-2.0
	F (m)	-1.6	-1.3	-1.5

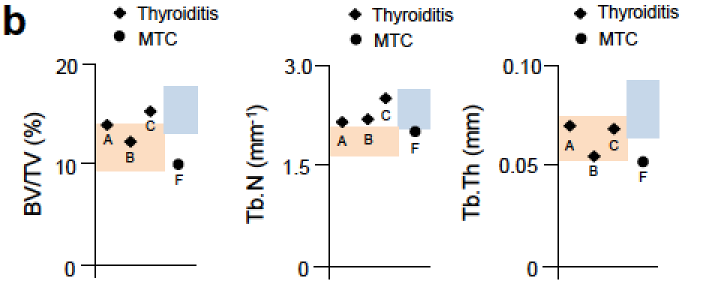

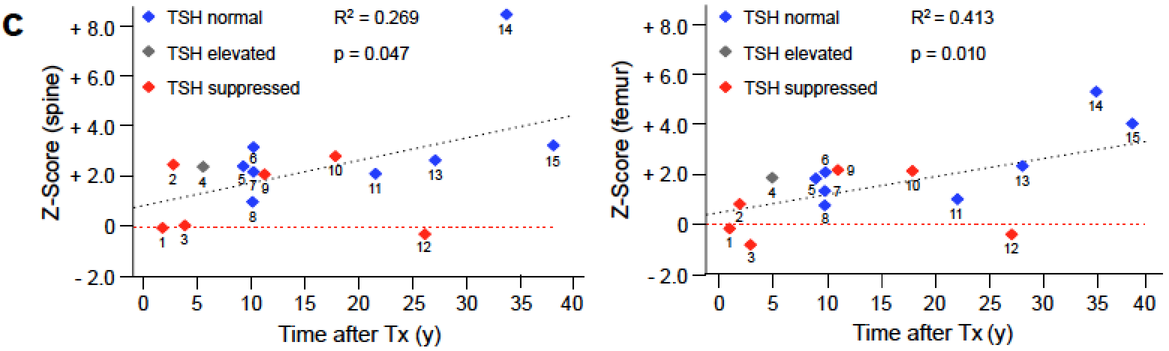

Supplementary Figure 6 | CT-deficiency is associated with high bone mineral density in humans. (**a**) DEXA Z-scores from different skeletal sites in three individuals with total thyroid insufficiency (thyroiditis) and in three patients suffering from medullary thyroid carcinoma (MTC). f=female, m=male. (**b**) HR-pQCT analysis of the left radius in the three subjects with thyroiditis and in one MTC patient (BV/TV, Bone volume per tissue volume; Tb.N., trabecular number, Tb.Th., trabecular thickness). Reference values derived from healthy young individuals aged 20 to 29 years are indicated in beige (women) or blue (men). (**c**) Linear regression representation of time after thyroidectomy (Tx) versus Z-score in spine and femur of thyroidectomized individuals. The average bone density of a person of the same age is indicated as the dotted red line.

	male		female	
Serum	*Calcr*$^{+/+}$	*Calcr*$^{-/-}$	*Calcr*$^{+/+}$	*Calcr*$^{-/-}$
Sodium [mM]	148±3	147±3	145±2	145±2
Potassium [mM]	4.2±0.3	4.2± 0.3	3.6±0.4	3.4±0.4
Chloride [mM]	108±2	107±3	109±2	109±1
Calcium [mM]	2.3±0.1	2.3±0.1	2.3±0.1	2.3±0.1
Phosphate [mM]	2.0±0.5	2.1±0.6	1.5±0.3	1.5±0.3

Supplementary Table 1 | Parameters of mineral homeostasis in 12 weeks old wildtype and *Calcr*-deficient littermates.
Data represent mean ± sd (n=12 mice per group).

	male		female	
Serum	*Calcr*$^{+/+}$	*Calcr*$^{-/-}$	*Calcr*$^{+/+}$	*Calcr*$^{-/-}$
Cholesterol[mM]	2.1±0.1	2.1±0.2	1.7±0.2	1.8±0.1
Triglyceride [mM]	1.3±0.3	1.3± 0.4	1.1±0.3	1.2±0.4
ALAT/GOT [U/l]	43±15	46±31	25±5	28±10
ASAT/GOT [U/l]	73±30	69±33	53±19	54±17
α-Amylase [U/l]	684±62	721±63	603±63	594±73
Glucose [mM]	17.3±2.2	16.0±2.0	14.4±2.6	15.3±2.0
LDH [U/l]	279±65	261±88	187±44	184±38
ALP [U/l]	81±9	96±17 *	131±11	147±12 *

Supplementary Table 2 | Parameters of metabolism and hepatic function in 12 weeks old wildtype and *Calcr*-deficient littermates.
*p<0.05 versus wildtype. Data represent mean ± sd (n=12 mice per group). P-values were assessed by two-tailed Student´s *t*-test.

	male		female	
Serum	*Calcr*$^{+/+}$	*Calcr*$^{-/-}$	*Calcr*$^{+/+}$	*Calcr*$^{-/-}$
Total protein [g/l]	46.4±2.1	46.5±2.0	49.5±2.1	48.5±1.1
Albumin [g/l]	23.6±1.6	23.7±1.3	27.3±1.6	27.3±1.1
Creatinine [µM]	19.9±2.0	19.1±2.9	16.6±1.6	18.5±1.0
Urea [mM]	11.6±1.4	11.6±1.0	12.5±1.7	12.9±1.0

Supplementary Table 3 | Parameters of kidney function in 12 weeks old wildtype and *Calcr*-deficient littermates.
Data represent mean ± sd (n=12 mice per group).

Patient	Sex	Age	Disease	CT (-42 ng/ml)	TSH (0.27-4.2 mU/l)	Ca (2.09-2.54 mmol/l)	Pi (0.77-1.54 mmol/l)	PTH (11-84 ng/l)
A	f	56	Thyroiditis	nd	2.51	2.41	1.01	53
B	f	61	Thyroiditis	nd	1.74	2.48	1.28	43
C	f	75	Thyroiditis	nd	3.05	2.26	0.9	32
D	f	68	MTC	357	0.37	2.23	0.91	98
E	m	32	MTC	400	5.65	1.86	0.79	47
F	m	79	MTC	37504	25.7	2.39	0.75	75

Supplementary Table 4 | Baseline characteristics of assessed individuals.

CT = Calcitonin, TSH = Thyroid-stimulating hormone, Ca = Calcium, Pi = inorganic Phosphate, PTH = Parathyroid hormone, MTC = Medullary thyroid carcinoma.

Patient	Sex	Age	Years after Tx	CT (-42 ng/ml)	TSH (0.27-4.2 mU/l)	Ca (2.09-2.54 mmol/l)	Pi (0.77-1.54 mmol/l)	PTH (11-84 ng/l)
1	f	44	1	nd	0.25	2.02	1.22	6
2	f	33	2	nd	0.2	2.19	0.79	32
3	f	51	3	nd	0.02	2.24	1.39	17
4	m	60	5	nd	25.64	2.36	0.96	9
5	f	49	9	nd	0.97	2.1	1.17	17
6	m	46	10	nd	0.308	2.39	0.76	22
7	m	65	10	nd	2.34	2.26	0.87	73
8	m	70	10	nd	1.16	2.05	0.9	3
9	f	61	11	nd	0.08	2.27	0.92	100
10	f	67	18	nd	0.032	2.24	1.1	4
11	m	80	22	nd	2.32	2.23	0.73	64
12	f	70	27	nd	<0.029	2.17	0.8	45
13	f	72	28	nd	0.68	2.33	1.03	13
14	f	77	35	nd	0.3	2.25	1.67	18
15	f	55	38	nd	1,5	2.32	1.13	33
I*	m	72	33	33.7	0.676	2.22	1	58.8
II*	f	81	33	nd	1.12	3.02	0.9	346

Supplementary Table 5 | Baseline characteristics of thyroidectomized individuals.

CT = Calcitonin, TSH = Thyroid-stimulating hormone, Ca = Calcium, Pi = inorganic Phosphate, PTH = Parathyroid hormone, MTC = Medullary thyroid carcinoma, Tx = Thyroidectomy. * excluded individuals due to detectable CT levels (I) or clinically apparent hyperparathyroidism (II).
